# Neuropsychological functioning and cognitive reserve in newly HIV diagnosed antiretroviral-naïve South African adults from peri-urban and informal settlements

**DOI:** 10.1371/journal.pone.0260260

**Published:** 2021-12-07

**Authors:** Kalpesh Narsi, Andrew Tomita, Suvira Ramlall

**Affiliations:** 1 Department of Psychiatry, University of KwaZulu-Natal, Durban, South Africa; 2 KwaZulu-Natal Research Innovation and Sequencing (KRISP), College of Health Sciences, University of KwaZulu-Natal, Durban, South Africa; 3 Centre for Rural Health, School of Nursing and Public Health, University of KwaZulu-Natal, Durban, South Africa; Universitas Indonesia Fakultas Kedokteran, INDONESIA

## Abstract

Despite lower incidences of HIV-associated dementia due to antiretroviral therapy, neuropsychological impairment (NPI) remains a persistent challenge in sub-Saharan Africa. Improving cognitive reserve (CR) can mitigate NPI, but there are few investigations on neuropsychological (NP) performance, and its association with CR in newly diagnosed ART-naïve HIV-positive individuals to inform early treatment strategies. A comprehensive battery of tests were administered to assess various NP domains (International HIV Dementia Scale [for memory, motor speed, psychomotor speed], Digit Span Test [for attention], Action Fluency Test [for language] and Clock Drawing Test [for executive/visuospatial function]), and CR (using Cognitive Reserve Index Questionnaire) among 211 newly diagnosed ART-naïve HIV-positive participants from two clinics that serve peri-urban and informal settlement communities in KwaZulu-Natal, South Africa. Regression models were fitted to assess the association between NP performance and CR controlling for socioeconomic and clinical factors. Test results revealed high levels of impairment across NP domains: language (96.7%), memory and psychomotor speed (82.5%), concentration (17.5%), executive function (15.2%) and visuo-spatial function (3.3%). Low CR and educational attainment were the only factors consistently associated with poor NP performance based on regression. High levels of impairment were found in certain NP domains in a relatively young group of newly diagnosed ART-naïve HIV-positive individuals. Residents of peri-urban and informal settlements face multitude of complex challenges in South Africa. An early multilevel intervention targeting clinical- (e.g. CR) and structural-level challenges (e.g. access to education) is needed for mitigating HIV-associated NPI and promoting long-term healthy living.

## Introduction

The life expectancy of people living with HIV (PLWH) in South Africa has been projected to increase to 70 years by 2030 due to the rollout of a national antiretroviral treatment (ART) programme [1]. This increased longevity brings in its wake a sub-population who would experience the combined effects of HIV and age-related cognitive decline, adding another dimension to the epidemic in South Africa [1]. HIV-associated neurocognitive disorders (HAND) not only impact on the quality of life, and social and occupational functioning, but may have a detrimental effect on adherence to ART in PLWH. Suboptimal adherence, in turn, may have serious adverse implications for both personal and public health, due to incomplete plasma viral suppression and the emergence of drug resistant strains of HIV [2, 3].

While the prevalence of HIV associated dementia (HAD) has decreased from the pre-ART era, milder forms of neuropsychological impairment (NPI), such as asymptomatic and mild neurocognitive impairments, have become more prevalent [4]. South African prevalence rates for any NPI is 25–80% [5–7]; however, most local studies have focused on populations that were previously eligible for ART (i.e., patients with lower CD4 counts) or those already receiving ART. Individuals with lower CD4 counts are more likely to have advanced NPI [8], while those exposed to ART may have impairment due to the effects of the medication and immune reconstitution [9]. There is little local data on the baseline neuropsychological function of patients with CD4 counts > 500 who are not on ART but may also have sub-optimum cognitive health due to HIV and other factors. With the eligibility for ART in South Africa now no longer dependent on the CD4 level, it becomes relevant to explore the role of socio-demographic, clinical and lifestyle factors, premorbid and early developmental variables in the cognitive health of PLWH.

Differences in cognitive health between the pre-ART and ART eras are not only reflected in the prevalence of the HAND syndromes, but also in the profiles of the cognitive domains affected. The fifth edition of the Diagnostic and Statistical Manual requires a diagnosis of neurocognitive disorders to be based on impairment in defined cognitive domains, but without the previously mandatory category of memory [10]. In the pre-ART era, HAND was characterized by impairments in motor skills, psychomotor speed and verbal fluency. However, in the ART era, impairments in memory and executive function are common, indicating a much earlier involvement of the subcortical and fronto-striatal brain areas [11]. There is little data on the profile of the various cognitive domains affected in the local HIV population [6, 12]; however, with the growing numbers of PLWH and their increased longevity, the pattern of NPI and its determinants become relevant for clinical, psychosocial and socio-economic reasons.

As with other neurocognitive disorders, numerous factors such as general physical health, depression, substance use, socio-demographic factors, economic status, lifestyle factors and cognitive reserve (CR), may influence the emergence and progression of NPI in PLWH [13, 14]. These represent either risk or resilience factors and, where modifiable, may offer therapeutic opportunities to mitigate added age-related cognitive decline.

CR refers to the ability of the brain to resist cognitive compromise in the face of neuropathology, based on the structural and biological makeup (“neural reserve”) and the compensatory, functional cognitive processes developed through neuroplasticity (“neural compensation”) [15]. This implies that the threshold for manifestation of NPI differs amongst individuals, depending on how efficient or resilient the cognitive processes are in compensating for the neural substrate affected by a disease process. The neural substrate is formed during foetal, childhood and adolescent development, and influenced by genetic expression and early childhood nutrition and stimulation. Functional cognitive processes are modifiable by the compensatory effects of education, intellectual stimulation, lifestyle habits and medical care, all of which maintain and protect neural reserve, and possibly increase neuroplasticity [16].

While the benefits of ART in reducing physical morbidity and mortality are undeniable, the impact of ART on milder forms of NPI is less robust. This may be due to certain ART’s suboptimal CNS penetration, their neurotoxicity, the immune reconstitution syndrome, and early neurocognitive and structural brain changes that occur during seroconversion, which are less responsive to ART [9, 17]. However, as treatment is necessary for survival, an assessment of cognitive ‘capital’ prior to ART exposure may be useful in understanding the likely impact of HAND in both young and ageing populations of PLWH. An assessment of CR and early NPI may assist in prognosticating long-term cognitive health and quality of life as well as offer insights on how this vulnerable group could maintain or improve their cognitive resilience. The aim of this study therefore was to describe the neuropsychological performance, and its association with CR, socio-demographic and clinical factors, of a clinical population of HIV positive adults prior to their initiation on ART.

## Method

Two community clinics in Durban, South Africa, were the chosen sites for a cross-sectional study. Patients attending these clinics reside in surrounding peri-urban and informal settlements. Between August and December 2018, all consecutive adult patients testing HIV-positive at the clinics’ routine universal test and treat program and meeting the study inclusion criteria were approached to participate in the study. The inclusion criteria were: age 18–60 years, a positive HIV diagnosis on either two rapid HIV tests or the ELISA, being ART-naïve, having a minimum education attainment level of Grade 5, and being able to read, write and speak English. Those excluded were participants with a history of a previous mental illness, and those with a medical illness or sensory deficit that precluded neuropsychological testing. The Biomedical Research and Ethics Committee, University of KwaZulu-Natal (Ref. No. BE205/18) and the Health Research Committee, KwaZulu-Natal Department of Health (NHRD Ref:KZ_201805_009) approved this study. Written informed consent was obtained from all participants.

After pre-initiation counselling and before being dispensed their prescribed ART, all participants were interviewed by the first author, a psychiatrist to assess eligibility for inclusion. To prevent inter-rater differences, a single data collector, a psychology honors student, interviewed all participants at both study sites using three sets of data tools. Firstly, biographical, clinical and lifestyle-related data was collected using a questionnaire, a structured diagnostic interview for depression, the Mini International Neuropsychiatric Interview, version 7.0.2 (M.I.N.I.), Depression Module [18], and clinical records. Secondly, CR was assessed using a semi-structured interview, the Cognitive Reserve Index Questionnaire (CRIq) [19]. The CRIq consists of 20 items grouped into three domains (education, working activity, and leisure time) that are considered mutually exclusive proxies for CR [20]. Each domain is scored based upon self- or informant-reported estimations of the number of years and frequency of various educational, occupational or leisure-related activities performed throughout the lifespan. The raw scores were entered in to a computational spreadsheet available from the author’s website (http://www.cognitivereserveindex.org) to calculate the age-adjusted final score (CRI-Total), which is categorized into five levels: low (<70), medium-low (70–84), medium (85–114), medium-high (115–130), and high (>130). Higher scores reflect a higher CR. The CRIq has not been validated locally, however, as it is not performance-based, the confounding effects of poor neurocognitive performance is mitigated when measuring CR. Thirdly, neurocognitive performance was assessed using a battery of neuropsychological tests: International HIV Dementia Scale (IHDS) [21], Digit Span Forward (DSF) and Digit Span Backward (DSB) [22], Action Fluency Test (AFT) [23], and Clock Drawing Test (CDT). The CDT was scored using the CLOX system to assess executive function (CLOX1) and visuospatial function (CLOX2) respectively [24]. The tests included in the battery were selected based on their use in previous local studies and their feasibility to administer in a resource-limited primary care setting. NPI for the various cognitive domains was defined as scoring below the cut-off values on respective neuropsychological tests (Table 1).

**Table 1:** Profile of neuropsychological tests used: Psychometric profile of tests.

Test	Neurocognitive domains tested	Cut-off	Sensitivity	Specificity	Validation
IHDS	Memory	≤10	62	76	South African HIV positive cohort [12]
	Motor speed				
	Psychomotor speed				
DSF	Attention	<5	42	93	South African HIV positive cohort [6]
DSB		<3			
AFT	Language	<15	76	84	HIV positive cohort [26]
CDT	Executive function (CLOX1)	<10	77	90	HIV positive cohort [25]
	Visuospatial function (CLOX2)	<12			

IHDS, International HIV Dementia Scale; DSF, Digit Span-Forward; DSB, Digit Span-Backward; AFT, Action Fluency Test; CDT, Clock Drawing Test.

Cut-off values for the IHDS, DSF and DSB were based on recommendations from local studies on a similar cohort to our participants [6, 12, 25, 26]; AFT and CDT cut-off values were based on normative data in a non-local, but clinically equivalent population [25, 27].

Statistical analysis was performed using STATA15, with the demographic and clinical characteristics of participants being summarized using means and standard deviations (SD) for continuous variables, or as proportions (%) for categorical variables. Performance on neuropsychological testing was analyzed using test scores as both continuous variables and categories based on cut-off values. A multiple regression model was generated to examine the relationship between demographic, CR and clinical risk predictors of neuropsychological performance. Converted z-scores from the neurocognitive tests were added and averaged to generate a composite cognitive score (CCS), which was used as the primary dependant variable in the model. Further regression analyses were done on each individual neuropsychological test, with a p-value of less than 0.05 being considered statistically significant.

## Results

### Socio-demographic and clinical profile

A detailed description of the clinical and cognitive reserve profile of participants has been reported in an earlier paper [28]. Of the 211 participants enrolled in the study, 149 (70.6%) were female and the mean age was 30.1 years (±7.8). All were African, with isiZulu being the primary language spoken by 97,2%. Grade 12 or higher was the highest level of education in 93 (44%) participants, and 118 (56%) had less than 12 years of education. Forty participants (19%) were formally employed, 64 (30.3%) were employed in the informal sector and 107 (50.7%) were unemployed.

Medical co-morbidity was reported by 26 (12.3%) participants, with pulmonary tuberculosis and asthma being the commonest conditions; 56 (26.5%) and 114 (54%) reported lifetime tobacco and alcohol use respectively. Of the 211, 25 (11.9%) met the criteria for current major depressive disorder. The majority (n = 128, 60.7%) had not suspected that they were infected with HIV until tested and 29 (13.7%) had suspected they were infected for more than 12 months (mean: 36.5; SD: 23.3) from the date of testing. The mean CD4 count of the 205 results that were traceable was 411 (SD: 277; range: 5–1433). The CR profile of 111 (52.6%) participants fell into low-medium category; almost half (n = 100, 47.4%) scored higher, placing them in the medium CR category, and none fell into the low or high CRIq range. A summary of the socio-demographic and clinical profile of the participants stratified by CR is presented in Table 2.

**Table 2:** Sociodemographic and clinical profile stratified by Cognitive Reserve Index (CRI) with p-values from univariate analysis.

Variable	Category	CRI category		Total	p-value
		Low-medium^a^ (n = 111)	Medium^b^ (n = 100)		
		n, (%)	n, (%)		
Age		M = 29	M = 31		
	18–29 years	60 (54.5)	50 (45.4)	110	0.74
	30–39 years	39 (52.0)	36 (48.0)	75	
	≥ 40 years	12 (46.2)	14 (53.8)	26	
Gender	Females	83 (55.7)	66 (44.3)	149	0.16
	Males	28 (45.2)	34 (54.8)	62	
Marital status	Single / Widowed	90 (52.0)	83 (48.0)	173	0.72
	Married / Co-habiting	21 (55.3)	17 (44.7)	38	
Educational attainment	≤ Grade 7	23 (100)	0 (0)	23	<0.01
	Grade 8–11	61 (64.2)	34 (35.8)	95	
	≥ Grade 12	27 (29.0)	66 (71.0)	93	
Employment status	Employed—formal sector	24 (60.0)	16 (40.0)	40	0.52
	Employed—Informal sector	31 (48.4)	33 (51.6)	64	
	Unemployed	56 (52.3)	51 (47.7)	107	
Substance Use (any)	No Substance Use	52 (54.7)	43 (45.3)	95	0.58
	Substance Use (any)	59 (50.9)	57 (49.1)	116	
Comorbid chronic illness	No	98 (53.0)	87 (47.0)	185	0.78
	Yes	13 (50)	13 (50)	26	
Depression status	Not depressed	99 (53.2)	87 (46.8)	186	0.62
	Major depressive disorder	12 (48.0)	13 (52.0)	25	
Suspected duration of HIV infection	Infection not suspected at time of testing	70 (54.7)	58 (45.3)	128	0.63
	Suspected infection in last 12 months	28 (51.9)	26 (48.1)	54	
	Suspected infection for > 12 months	13 (44.8)	16 (55.2)	29	
CD4 count (n = 205)		M = 401	M = 422		
	< 200	27 (58.7)	19 (41.3)	46	0.52
	200–499	50 (53.2)	44 (46.8)	94	
	≥ 500	31 (47.7)	34 (52.3)	65	

CRI, Cognitive Reserve Index.

^a^ Cognitive Reserve Index Questionnaire (CRIq) score: 70–84;

^b^ CRIq score: 85–114.

### Performance on neuropsychological tests

Table 3 summarises participants’ performances across the different cognitive domains tested. The majority (82.5%) evidenced NPI on the IHDS. Performance on AFT was also poor, with 96,7% scoring below the recommended cut-off of < 15. More participants showed impairment on DSB (18%) than DSF (1.4%). On the CDT, just over 15% scored below the cut-off for executive functioning and impairment on visuospatial functioning was marginal (3.3%).

**Table 3:** Performance on neuropsychological tests.

Cognitive domain	Test	Mean	Range	SD	Cut off value	Below cut-off	Above cut-off
						n (%)	n (%)
Psychomotor speed and memory	IHDS Total	8.42	3–12	1.96	≤ 10	174 (82.5)	37 (17.5)
	Motor speed	2.9	1–4	0.79			
	psychomotor speed	2.5	0–4	1.11			
	Memory	3.03	0–4	1.01			
Attention and Concentration	DSF	5.18	3–8	0.98	<5	3 (1.4)	208 (98.6)
	DSB	3.27	2–6	0.85	<3	38 (18)	173 (82)
Language	AFT	8.67	3–19	2.69	<15	194 (96.7)	7(3.3)
Executive Function	CDT—CLOX1	11.9	4–15	2.32	<10	32 (15.2)	179 (84.8)
Visuospatial Function	CDT—CLOX2	13.9	4–15	1.31	<12	7 (3.3)	203 (97.7)

IHDS, International HIV Dementia Scale; DSF, Digit Span-Forward; DSB, Digit Span-Backward; AFT, Action Fluency Test; CDT, Clock Drawing Test.

### Socio-demographic and clinical predictors of neuropsychological test performance

A multiple regression model was generated to investigate the association between clinical and socio-demographic factors, CR (CRI-Total) and neuropsychological performance (Table 4).

**Table 4:** Regression model investigating adjusted associations between socio-demographic and clinical factors and neuropsychological performance.

Socio-demographic clinical predictors	Reference	Variables	Composite Cognitive Score			IHDS			DSF			DSB			AFT			CLOX1			CLOX2		
			B	SE	p	B	SE	p	B	SE	p	B	SE	p	B	SE	p	B	SE	p	B	SE	p
Gender	Female	Male	-0.72	0.54	0.19	-0.32	0.28	0.25	-0.19	0.16	0.24	-0.05	0.14	0.71	-0.04	0.40	0.92	-0.12	0.37	0.75	-0.30	0.22	0.16
Age category	18–29 years	30–39	-0.59	0.52	0.26	-0.79	0.28	**< .01**	0.10	0.15	0.50	-0.03	0.13	0.84	-0.38	0.39	0.33	-0.32	0.37	0.38	0.03	0.21	0.88
		40+	-0.88	0.78	0.26	-0.92	0.41	**0.03**	0.05	0.23	0.84	-0.07	0.20	0.72	-0.27	0.58	0.64	-0.72	0.55	0.19	0.04	0.32	0.89
Educational attainment	< = Grade 7	Grade 8–11	2.31	0.81	**< .01**	0.66	0.42	0.12	0.55	0.23	**0.02**	0.41	0.20	**0.04**	-0.79	0.60	0.19	1.61	0.56	**< .01**	0.68	0.33	**0.04**
		Grade 12+	2.88	0.88	**< .01**	0.36	0.46	0.43	0.70	0.25	**< .01**	0.53	0.22	**0.02**	0.38	0.65	0.56	1.86	0.61	**< .01**	0.55	0.36	0.12
Employment status	Unemployed	Formal sector	-0.33	0.66	0.62	-0.33	0.35	0.35	-0.09	0.20	0.63	0.01	0.17	0.97	-0.94	0.50	0.06	0.20	0.47	0.66	0.24	0.27	0.37
		Informal sector	-0.14	0.61	0.82	-0.01	0.32	0.97	-0.17	0.18	0.35	-0.03	0.16	0.87	-0.21	0.46	0.65	0.23	0.43	0.59	0.06	0.25	0.80
Substance Use	No	Yes	0.66	0.46	0.15	0.60	0.24	**0.01**	0.04	0.14	0.76	0.20	0.12	0.08	-0.01	0.34	0.98	0.15	0.32	0.65	0.01	0.19	0.95
Comorbid chronic illness	No	Yes	0.46	0.70	0.52	0.91	0.37	**0.01**	0.12	0.21	0.57	0.04	0.18	0.84	-1.53	0.53	**< .01**	0.11	0.49	0.83	0.46	0.28	0.11
Significant Head injury	No	Yes	-1.03	0.97	0.29	-1.24	0.51	**0.02**	-0.16	0.29	0.59	-0.36	0.25	0.15	-0.12	0.73	0.87	0.14	0.68	0.83	0.22	0.39	0.57
Depression Status	No MDD	MDD	0.37	0.68	0.58	0.03	0.36	0.93	0.02	0.20	0.93	-0.20	0.17	0.25	0.30	0.51	0.55	0.54	0.47	0.25	0.29	0.27	0.29
CRIq score	70–84	85–114	2.40	0.50	**< .01**	1.42	0.27	**< .01**	0.20	0.15	0.17	0.32	0.13	**0.01**	1.41	0.38	**< .01**	0.59	0.35	0.09	0.41	0.20	**0.05**
Suspected Infection duration	Infection not suspected	Infected ≤12 months	0.13	0.53	0.80	0.18	0.28	0.52	0.05	0.16	0.77	-0.07	0.13	0.58	0.07	0.40	0.87	0.02	0.37	0.95	0.06	0.21	0.79
		Infected > 12 months	-0.14	0.69	0.84	0.16	0.37	0.67	-0.05	0.20	0.81	0.00	0.17	0.98	1.02	0.52	0.05	-0.51	0.48	0.30	-0.44	0.28	0.12
CD4 category	<200	200–499	0.60	0.58	0.30	0.61	0.31	**0.05**	-0.01	0.17	0.97	0.18	0.15	0.23	0.50	0.43	0.25	-0.14	0.41	0.73	-0.04	0.23	0.85
		500+	0.06	0.64	0.92	0.04	0.34	0.90	-0.10	0.19	0.61	-0.05	0.16	0.77	1.00	0.48	**0.04**	-0.21	0.45	0.64	-0.11	0.26	0.67

IHDS, International HIV Dementia Scale; DSF, Digit Span-Forward; DSB, Digit Span-Backward; AFT, Action Fluency Test; CLOX1, Clock Drawing Test—executive function score; CLOX2, Clock Drawing Test—visuospatial function score; MDD, major depressive disorder; CRIq, Cognitive Reserve Index Questionnaire.

**Fig 1:**
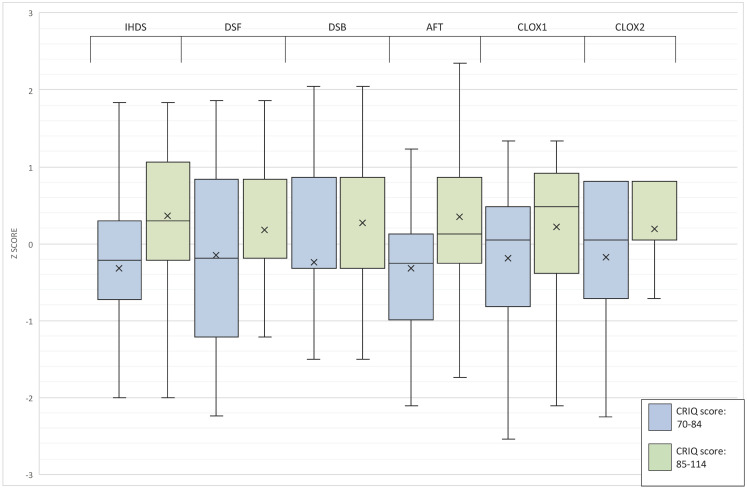
Box and whisker plot of averaged neuropsychological test performance z scores according to Cognitive Reserve Index categories.

Gender and employment status were not associated with changes in performance, and those older than 30 years had significantly lower scores on the IHDS (p<0.05), but not on other neuropsychological tests or CCS. Educational attainment was significantly associated with better CCS (p<0.01), but not on the IHDS. Regarding clinical predictors, a lifetime history of any substance use and a comorbid chronic medical condition were both associated with higher scores on IHDS (p<0.05) and a history of significant head injury was associated with lower IHDS scores (p<0.05), while other cognitive domains and CCS were not associated with these variables. The presence of major depressive disorder and the suspected duration of infection were not significantly predictive of performance in any cognitive domain. Compared to participants with CD4 counts of less than 200, those with higher counts had higher IHDS (p = 0.048) and AFT scores (p = 0.037). After controlling for socio-demographic and other clinical co-variates, higher CRI-Total was significantly predictive of higher CCS and better performance domain-specific neuropsychological tests (Fig 1).

## Discussion

The study described the neuropsychological test performance and its association with CR, socio-demographic and clinical factors in a local sample of ART-naïve, newly diagnosed HIV positive adults. The results demonstrated the high burden of baseline NPI in PLWH prior to their initiation on ART, and highlighted the significant association between NPI and other socio-demographic factors, such as age and education, but particularly CR.

The socio-demographic profile of the study participants: two-thirds female, average of 30.1 years, predominantly single and unemployed, is consistent with the trend of newly HIV-diagnosed adults in South Africa [29]. Exclusion bias may account for the higher educational attainment of the sample (89.1% having some secondary education and higher) compared to other local studies on this population [29, 30]. However, the educational level and employment status of the participants mirrors the local demographics [31], and the socio-demographic profile of our clinical sample is also reflective of the cohort of PLWH in the community.

### Neuropsychological test performance

The majority of participants displayed impairment in memory, motor and psychomotor function, as evidenced by the large number who screened positive on the IHDS (82.5%). Our results replicate the findings of earlier local studies [7, 32], but is much higher than the prevalence reported by Goodkin et al. (43%) and Joska et al. (67,8%) [12, 33]. However, Joska et al. noted that construct validity was not established for the translated neuropsychological tests used in their study on a Xhosa population in Cape Town. This resulted in poorer performance on their battery compared to the IHDS, potentially increasing their yield of false negatives.

We found a substantially higher degree of impairment in verbal fluency compared to the literature, this being the most frequently identified expressive language deficit estimated to occur in approximately 40–60% of individuals with HAND [34]. While AFT may be confounded by psychomotor speed, executive function, educational and cultural factors, it may be more sensitive than other fluency tests, such as letter and category fluency. The latter tests evoke noun generation, which is primarily dependent on the temporo-parietal networks and semantic memory stores. By contrast, verb generation (as used in AFT) is associated with activity in the frontal systems, executive functions and motor planning systems, all areas known to be affected early in HAND [34]. Therefore, the utility of AFT as a screening tool in the local context merits further investigation, with local normative data needed to set the cut-off for AFT at one standard deviation, as recommended by Woods et al. [26].

Attentional deficits in our sample were consistent with findings from local data and support the recommendations by Singh et al. in considering DSF and DSB as suitable supplements to the IHDS in the clinical setting [6]. As attention is predictive of medication adherence in PLWH, including its assessment as a component of neurocognitive screening may have public health advantages [34].

Executive dysfunction in our sample was much lower than reported in other African studies [35, 36], with local literature recommending the Trail Making Test—Part B (TMT-B) as a suitable supplement to the IHDS for screening [6, 33]. However, while routine screening of executive function using the TMT-B requires pre-printed sheets, the CDT requires no resources to administer in a primary care setting. There is also evidence for its utility as a screening tool for executive functioning in the local aged population [37]. Robbins et al. noted that PLWH performed significantly worse than controls on the CDT component of the Montreal Cognitive Assessment (MOCA) [7], with the CDT’s utility as a stand-alone executive function screening tool in this population warranting consideration. A systematic review of executive dysfunction found that discrete domains of executive function may be differently affected in PLWH, which may explain the discrepant prevalence of executive dysfunction with different instruments [38].

Visuospatial cognition is important for driving and engagement in physical and leisure activities [39], which in turn impacts on CR. Therefore, impaired visuospatial cognition may ignite a vicious cycle of compromised recreational activities, lower CR and poorer neurocognitive function in multiple domains. Visuospatial cognition was initially believed to be unaffected in HAND, but studies support the likelihood that subtle deficits exist in spatial cognition, which may include problems with understanding, manipulating and integrating visual stimuli [34]. The CLOX2 score from the CDT assesses basic visuo-constructive function and may be less sensitive to subtle deficits. A local study noted that both PLWH and controls performed equally poorly on the cube drawing component of the MOCA [7]. This finding, and the low proportion of visuospatial impairment in our participants (n = 7, 3.3%), highlight the need for further investigation of appropriate tests to assess visuospatial cognition in the local PLWH population.

### Ageing and cognition

The finding that older participants had significantly lower scores on the IHDS is in keeping with the literature [5, 33]. Premature ageing by 10–15 years occurs in PLWH by two possible mechanisms: acceleration of the ageing process by HIV, and accentuation of ageing through comorbid medical illnesses [40]. Our results indicate that age was significantly associated with NPI, independent of comorbid illness. The association remained significant when controlling for CR, despite increasing age correlating with higher CR. NPI in the ageing HIV population may reflect the acceleration of the senescent process, with a South African study finding shorter telomere lengths and greater expression of CDKN2A (a marker of cellular senescence) in PLWH compared to controls [41].

### Depression and cognition

The 11.9% prevalence of current major depressive disorder (MDD) in our sample was more than twice the expected 12-month prevalence of 4.9% in the South African population [42], but considerably lower than the 30–40% reported elsewhere [43, 44]. This may be due to our participants being recruited early in the course of their infection, with 60.7% not suspecting that they were infected at the time of testing. Hence, the psychological impact of an HIV diagnosis, the duration of infection, and the neuropsychiatric side-effects of ART, all of which are established risk factors for MDD in PLWH [43], may have had a minimal contribution in our study. The presence of MDD was also not independently associated with impairment in any specific cognitive domain or overall. However, the long-term effects of MDD on cognition and CR requires further research, as identification and treatment of MDD, which is a recognised risk factor for NPI in the aged, is an important strategy to improve CR, preserve cognition and prevent decline [43].

### Cognitive reserve and neuropsychological test performance

The positive association between CR and CCS in our sample adds to the growing evidence that PLWH with lower CR are at higher risk for NPI and symptomatic HAND (MND and HAD), independent of depression and HIV staging [45]. Furthermore, our finding that CR was independently predictive of impairment in all the discrete cognitive domains is congruent with the findings of Stern et al. In their study, PLWH with low CR had significantly greater deficits in the domains of attention, information processing speed, verbal memory, executive functioning, and visuospatial performance, which are the primary domains affected by HAND [46].

CR is influenced by intelligence quotient (IQ), educational attainment and occupation, as well as intellectual, social and physical activities, with many studies having used these as proxies for measuring CR [47]. The CRIq used in our study is one of few CR tools that is not performance-based and excludes measures of IQ [17], thus mitigating the confounding effects of NPI on our measure of CR. Educational attainment was found to influence CR and was predictive of better performance in attention and executive function in our study. Therefore, our finding that CR was significantly associated with better performance on memory, psychomotor processing and visuospatial tasks, independent of educational and occupational attainment, may highlight the contribution of leisure and social activities in preserving certain cognitive functions over others. This is relevant in low-resourced settings, where educational opportunities are often limited, youth unemployment is high, and poverty further exacerbates poor access health care, cognitively stimulating vocations and activities.

The value of leisure activities in building CR is evidenced by pre-clinical data showing that a stimulating environment and regular exercise promote neurogenesis in the dentate gyrus and regulate factors such as brain derived neurotropic factor that increase neuronal plasticity [48]. A review by Quigley et al. found that physical activity may preserve and improve cognitive function in PLWH, with possibly greater benefits to the domains of memory and psychomotor processing [49]. Our results suggest that these findings may also be relevant locally, with the role of exercise and leisure as neuroprotective factors in PLHW warranting further study.

### Limitations

We acknowledge the several limitations of our study. The demonstrated cross-sectional associations preclude causation being inferred. Factors influencing CR may also influence performance on cognitive tasks; longitudinal studies to assess the neuroprotective influence of CR, especially the contributory role of physical and leisure activities, are therefore necessary. Our study population consisted of those presenting for HIV testing at a public sector clinic, which may represent a biased sample of individuals with health-seeking behaviour, who were able to consent to ART initiation, and therefore having better cognitive capacity; this makes it difficult to generalise the results to a community population. As substance use was not quantified in our study, the finding that current, self-reported substance use was associated with improved neuropsychological performance (NP) should be interpreted with caution. The literacy requirements of the research tools that were used limited the participants to English-speakers with a Grade 8 or higher education level, which may have confounded the measures of CR and neuropsychological performance. Furthermore, we acknowledge that some tools used in this study to measure NPI were screening tools rather than formal neuropsychological tests, and local normative data is unavailable for many of these. Finally, although the CRIq has not been validated locally, it is not performance-based; its activity items are congruent with local leisure activities [50], thus minimising potential cultural bias. We acknowledge that while the lack of normative data calls for our sample’s NP and CR category ranges to be applied with caution in our populations, inferences may still be made on associations with CR variability and NP.

## Conclusion

Unlike other degenerative neurocognitive disorders, HAND is not invariably progressive, and therefore efforts at intervention should be encouraged, especially in the context of the high local disease burden. ART has thus far been the mainstay of treatment of NPI in PLWH, with systematic reviews and longitudinal studies indicating that it is only effective in targeting limited cognitive domains and more severe impairment [15, 51]. Despite treatment with ART, milder NPI persists and may even be progressive, independent of viral suppression, immune recovery and the central nervous system penetrability of the chosen ART regime [52]. There is therefore a need to develop non-pharmaceutical strategies for the primary prevention of HAND. Holistic management programmes that promote cognition-enhancing physical and social activities may not only play a role in prevention, but growing evidence also affirms their therapeutic value [49].

Notwithstanding the limitations, this is the first study, to our knowledge, that includes CR amongst the numerous factors associated with NPI in a South African sample of PLWH. With the incumbent National Health Insurance’s prioritization of prevention, a shift is required in research and practice as to how neuropsychological functioning may be preserved. Our findings contribute to the emerging literature on the role of CR in preventing NPI and suggests there is a strong association between the two. A comprehensive, holistic, multi-sectoral approach that promotes medical and non-medical interventions, and focuses on education, employment and leisure activities is necessary to build cognitive resilience. Therefore, activities that promote CR merit further exploration as a public health preventative strategy for a growing and greying population of PLWH.
